# Initiation, Evolution, Excellency and the “Sacred rigor of surgery”

**Published:** 2014

One of the great founders of the Romanian surgery school, Professor Ion Juvara was honored in the continuity tradition of recognizing the value of the ancestors in the enthusiastic and full of warmth environment of the beginning of the academic year in the Aula Magna of “CAROL DAVILA” UNIVERSITY OF MEDICINE AND PHARMACY in Bucharest.

**Fig. 1:**
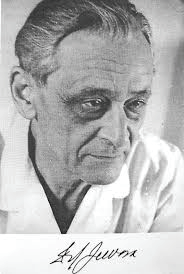
Professor Ion Juvara – portrait

The laborious activity of Professor Ion Juvara was abundantly and excitedly presented by famous personalities of the University: Acad. Ioanel Sinescu, Rector of “Carol Davila” University of Medicine and Pharmacy, Prof. Mircea Beuran, MD, Head of the Surgery Clinic of the Emergency Hospital in Bucharest and the Head of the Surgery Department, Prof. Traian Patrascu, MD, Head of the Surgery Clinic of “Cantacuzino” Hospital, Prof. Dan Sabau, MD, from “Lucian Blaga” University in Sibiu, famous followers of the exceptional surgeon and also Prof. Radu Palade, MD, a famous surgeon, son of the great scientist George Emil Palade, who had the chance of meeting Ion Juvara when he was only 3 years old. The ceremony was admirably completed by the memories full of emotion and nostalgia of the great surgeon’s family who was also present at the event. 

Just as the speakers underlined, Prof. Ion Juvara, MD, was a true personality, who has unmistakably marked the Romanian surgery field of the second half of the last century. Born 100 years ago in Bucharest, he enrolled, when still being young, on a singular trajectory of performance; occupying the first position in all the professional competitions he took part in.

**Fig. 2:**
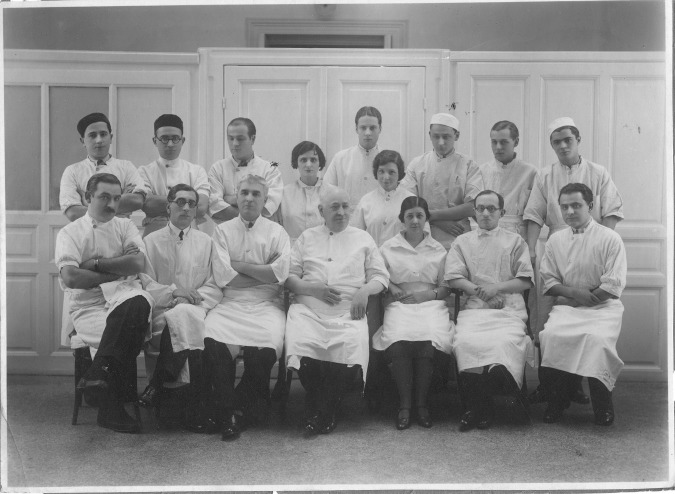
Professor Ion Juvara and his fellow doctors

Among the professors who have marked the professional trajectory of young Ion Juvara, there are two great personalities of the Romanian medical school: Francisc Reiner and Nicolae Hortolomei, and among the friends from his youth – the future laureate of Nobel Prize, George Emil Palade.

As the speakers confirmed, the scientific level, the impeccable technique, and last, but not least, the courage based on the objective appreciation of his own possibilities, allowed professor Juvara to approach the pioneering fields of surgery in that period of time: the first surgeries on the thyroid gland made together with professor Hortolomei, the first thymectomies, the new interventions on the thorax or heart, being only few of the paths opened by this great surgeon’s talent and visionary spirit.

**Fig. 3:**
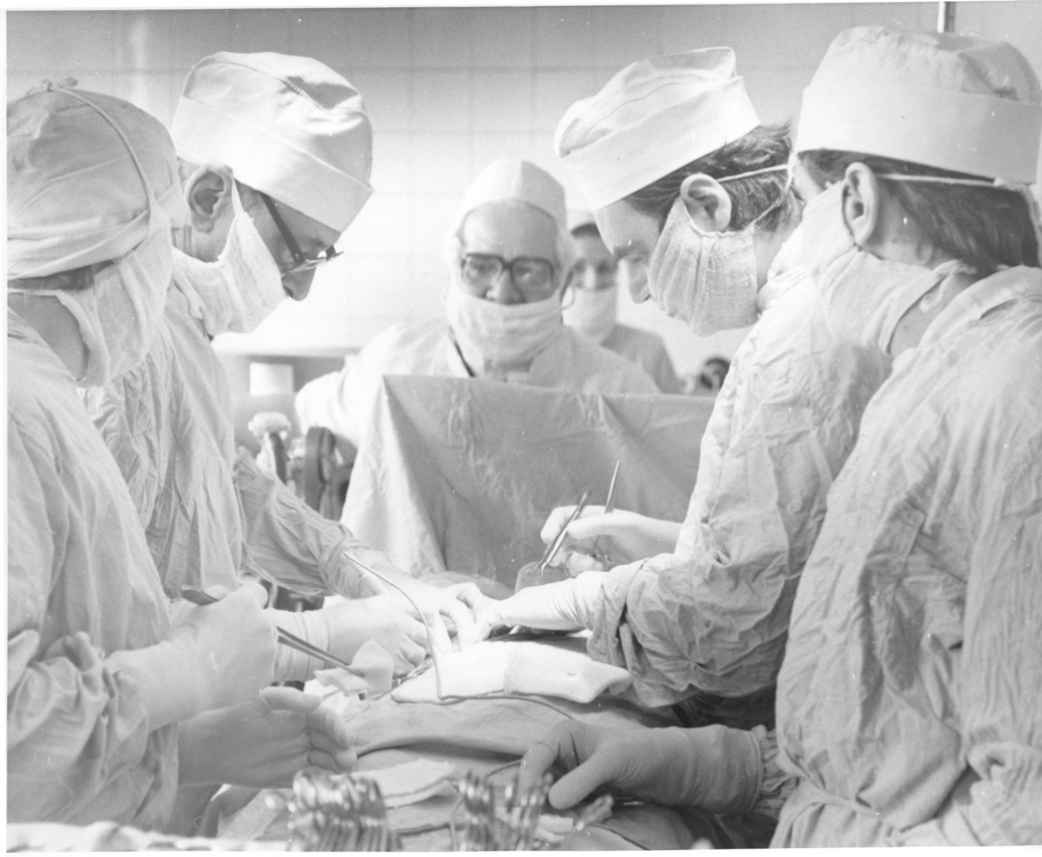
Professor Ion Juvara while performing a surgery

His prodigious scientific activity includes 460 presented or published papers. The main scientific contributions, which have remained in history as true “milestones” in the field of surgery are the ones concerning the thyroid gland, organic hypoglycemia due to pancreatitis or the treatment of myasthenia gravis by using thymectomy. The studies on reinterventions in gastric surgery and in extrahepatic biliary tracts have also been of great interest until present, his preoccupation for a clear and an in-depth study of knowledge in a field so sensitive and difficult, of imperfections and surgical failures being remarkable. 

**Fig. 4:**
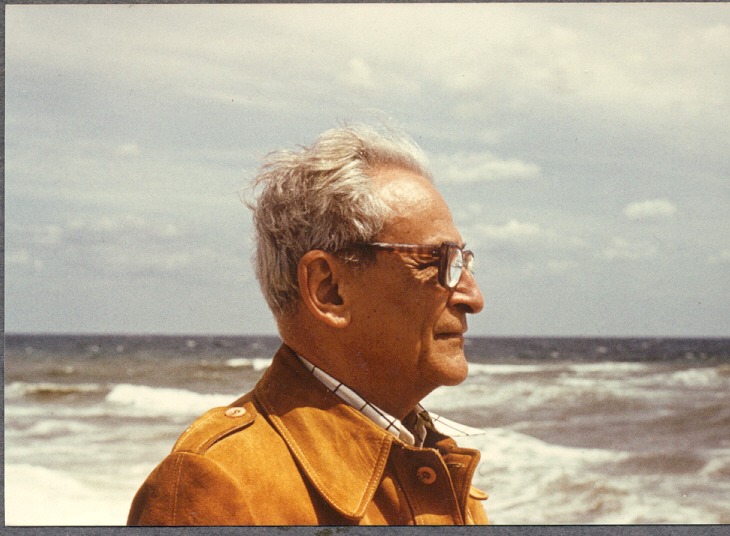
Professor Ion Juvara contemplating

In 1996, at the venerable age of 83 years old, at the end of a fulfilled road both from the family point of view and from a professional one, professor Ion Juvara stepped quietly out of life, entering the legend.

And the legends never die! 

**Executive Editor** **Assoc. Prof. Dr. Eng. Victor Lorin Purcarea**

